# The American and Colonial Journals: Pathology, Practical Medicine, and Therapeutics

**Published:** 1840-04

**Authors:** 


### PATHOLOGY, PRACTICAL MEDICINE, AND THERAPEUTICS.

*Case illustrative of the Etiology of Spontaneous Amputation of the Limbs of the Fœtus in Utero.* By A. H. BUCHANAN, M.D., of Columbia, Tennessee.

IN the month of February last I was called in haste to the country, about three miles, to see a negro woman who was said to be suffering from severe pain in the back, and uterine hemorrhage. She was the mother of ten children, aged about forty years, and had miscarried three or four times. On my arrival, I found she had aborted, and that the uterine hemorrhage had ceased. Upon an examination of the fœtus, which was between three and four months old, and perfectly formed, except a considerable flattening of the head laterally, I found the umbilical cord twisted about the thigh and neck in the following manner: the cord passes from the umbilicus under the right thigh, just above the knee-joint, and continuing completely around it, passes under itself, and ascends in front of the chest to the right side of the neck, around which it twines twice, or rather twice and a half, so that two coils are seen in front of the neck and three behind; it then passes in front of the left shoulder to the placenta. From the compressed appearance of the cord opposite the left shoulder I think it passed under the left armpit to the placenta. Thus circumstanced, it is evident that any efforts made by the child to extend the thigh tightened the cord about the neck and also about the thigh, as well as dragged upon its umbilical extremity, and obstructed the circulation. The same effects also are produced by extending back the head; but in this last action, the placental extremity of the cord is immediately pulled upon. It is very fair to conclude that the fœtus thus situated came to its death either from the compression of its throat by the cord or from its obstructed circulation, or from both; and that the abortion was a consequence of its death. At many points where the cord twists upon itself it is very much compressed, or rather atrophied. But the object of communicating this case is to call attention to the effects produced upon the thigh by the twisting of the cord around it. It may be seen by any present, that at the point of compression only the integuments intervene between the cord and bone, all the other parts having disappeared; but the limb below the ligature appears as fully developed as its fellow, and the integuments immediately under the ligature appear sound. Now it is highly probable, had the child lived to its full time, the leg would have been amputated by the process of absorption carried on in consequence of the pressure of the cord around the limb, and that the opposite surfaces would have healed, as is usual in such cases, during the process of amputation; the limb below the ligature retaining its vitality by its connexion with the integuments, they being the last parts to give way during the amputation; that the leg below the knee-joint would have been more or less atrophied before its complete separation is almost certain.

*American Journal of Med. Sciences.* No. xlviii.

---

*On the Causes of the Acute Diseases of European Soldiers in India.* By J. MOUAT, M.D., Surgeon of H. M. 13th Light Dragoons.

[THE following Table constitutes only a small part of a most elaborate and valuable report on the health of the 13th Light Dragoons, published in the first number of the new "Madras Quarterly Medical Journal," which has not yet reached us. Our readers will perhaps be surprised at the very striking part played by *cold* in this tropical etiology :]

*Table of the Causes or assigned Causes of Acute Disease in the 13th Light Dragoons, from 1831 to 1838, inclusive.*

| | |
|---|---|
| Who impute their disease to cold caught on line guard and picquet, by sleeping under the trees or in the open air, or in the sick lines and exposed to a current of air . . . . . . . . | 259 |
| Who impute their disease to cold caught on their posts whilst sentry at the sick lines, hospital, paymasters, and the barrack guards, or by going round with the relief . . . . . . . . | 95 |

| | |
|---|---|
| Who impute their disease to cold caught on barrack, mess-house, paymasters and choultry guards, by sleeping in the verandas or open air, and exposed to a current of air . . . . . . . | 125 |
| Who impute their disease to cold caught by sleeping opposite windows or doors in the barracks that were left open . . . . | 287 |
| Who impute their disease to cold caught by being out with the band of the regiment at balls . . . . . . . | 4 |
| Who impute their disease to cold, but are unable to particularize how . | 261 |
| Who impute their disease to cold caught by getting wet in the rain when on barrack guard, line guard, parades, sentry, shoeing horses, at lines, field exercise, whilst going to the privy, bazar, or by getting the soles of their feet wet . | 197 |
| Who impute their disease to cold caught by the changes of the weather . | 43 |
| Who impute their disease to cold caught by neglecting to put on their flannel jackets . . . . . . . | 6 |
| Who impute their disease to cold caught by drinking cold water or buttermilk, or by bathing in cold water when heated . . . . | 55 |
| Who impute their disease to cold caught by sleeping on the floors of the barracks on account of being annoyed by bugs . . . . | 2 |
| Who impute their disease to cold caught by sleeping in the open air . | 4 |
| Who impute their disease to cold caught by sleeping uncovered in the barracks | 16 |
| Who impute their disease to cold caught on the march from Poonamallee . | 5 |
| Who impute their disease to cold caught during their confinement in the barrack guard and cells . . . . . . | 11 |
| Who impute their disease to cold caught by being a long time in water to get out a boat . . . . . . . | 2 |
| Who impute their disease to exposure in the sun whilst on barrack guard, line guard, shoeing horses, sentry, on duty superintending horsekeepers at work, piling up hay, going to and returning from church, &c. . . | 62 |
| Who impute their disease to diet, as having eat mulligatawny and rice, country greens, cold meat, boiled and mashed potatoes, fat bacon, milk and bread, eggs, curry and rice, jackfruit, oranges, cucumbers, drinking hot tea, milk, and cold water . . . . . . . | 26 |
| Who impute their disease to jerks or falls from their horses whilst at riding school, going to water, field exercise, or out in watering order, to the tightness of their full dress, to costiveness, to rubbing in mercurial ointment on mangy horses, to violent exertions, &c. &c. . . . . | 59 |
| Whose diseases were caused by intemperance . . . . | 105 |
| Who know no cause for their disease but have been drinking . . | 14 |
| Who know no cause for their disease . . . . . | 1658 |
| Diseases readmitted from the above from changing to other acute affections . | 98 |
| Grand Total . | 3394 |

*India Journal.* No. xlii.

